# Prevention of Asbestos-Related Disease in Countries Currently Using Asbestos

**DOI:** 10.3390/ijerph13050494

**Published:** 2016-05-12

**Authors:** Daniela Marsili, Benedetto Terracini, Vilma S. Santana, Juan Pablo Ramos-Bonilla, Roberto Pasetto, Agata Mazzeo, Dana Loomis, Pietro Comba, Eduardo Algranti

**Affiliations:** 1 Environment and Primary Prevention, Istituto Superiore di Sanità, Rome 00161, Italy; roberto.pasetto@iss.it (R.P.); pietro.comba@iss.it (P.C.); 2 Professor of Biostatistics, University of Turin (Now Retired), Turin 10124, Italy; benedetto.terracini@fastwebnet.it; 3 Instituto de Saude Coletiva, Universidade Federal da Bahia, Salvador 40110-040, Brazil; vilma_santana50@hotmail.com; 4 Departamento de Ingeniería Civil y Ambiental/Department of Civil and Environmental Engineering, Universidad de los Andes, Bogotá 110231, Colombia; jramos@uniandes.edu.co; 5 WHO Collaborating Centre for Environmental Health in Contaminated Sites, Istituto Superiore di Sanità, Rome 00161, Italy; 6 Department of History and Cultures, University of Bologna, Bologna 40126, Italy; agata.mazzeo2@unibo.it; 7 International Agency for Research on Cancer, Lyon 69372, France; LoomisD@iarc.fr; 8 Serviço de Medicina, FUNDACENTRO, São Paulo 05409-002, Brazil; eduardo@fundacentro.gov.br

**Keywords:** asbestos, prevention, Latin America, international cooperation, asbestos-contaminated communities

## Abstract

More than 40 years of evaluation have consistently confirmed the carcinogenicity of asbestos in all of its forms. This notwithstanding, according to recent figures, the annual world production of asbestos is approximatively 2,000,000 tons. Currently, about 90% of world asbestos comes from four countries: Russia, China, Brazil and Kazakhstan; and the wide use of asbestos worldwide represents a global threat. The purpose of this paper is to present a review of the asbestos health impact and to discuss the role of epidemiological investigations in countries where asbestos is still used. In these contexts, new, “local” studies can stimulate awareness of the size of the problem by public opinion and other stakeholders and provide important information on the circumstances of exposure, as well as local asbestos-related health impacts. This paper suggests an agenda for an international cooperation framework dedicated to foster a public health response to asbestos, including: new epidemiological studies for assessing the health impact of asbestos in specific contexts; socio-cultural and economic analyses for contributing to identifying stakeholders and to address both the local and global implications of asbestos diffusion; public awareness on the health and socio-economic impact of asbestos use and banning.

## 1. Foreword

The present paper summarizes the presentations and discussion of the symposium “Prevention of asbestos-related disease in Latin America: reducing inequalities by international cooperation” held within the International Society for Environmental Epidemiology (ISEE) 2015 Conference in São Paulo (Brazil), also taking into account the issues raised in the same days at the workshop “Occupational asbestos exposure and health in Brazil: an interdisciplinary project” held at São Paulo University.

Brazil today can be regarded as a fault line characterized by a direct confrontation between pro- and anti-asbestos forces, pushing, respectively, for continuing extraction, use and export of the mineral on one side and asbestos banning on the other. Brazil, especially, and Latin America at large well represent at the regional level that the on-going global clash on asbestos impacts society at large, including industry, labor, public health, urban planning, media and courtrooms. In this frame, the scientific community is required to stand and speak out clearly.

The purpose of this paper is to present a review of the asbestos health impact, to illustrate the arguments in favor and against continuing asbestos use, to discuss the role of epidemiological investigations in countries where asbestos is still used, to examine in depth the current situation in Brazil, including the civil struggle of asbestos victims, and finally, to suggest an agenda for international cooperation in fostering the public health response to asbestos.

## 2. The Carcinogenic Risk of Asbestos: Evaluation and Public Health Implications

Evidence about the carcinogenicity of asbestos has been accumulating since the first part of the 20th Century. Suspicions that lung cancer might be associated with exposure to asbestos were first reported in the USA and the UK in the 1930s, followed about a decade later by reports of pleural tumors associated with asbestos [1].

The first epidemiological studies of cancer risk in relation to exposure to asbestos were reported in the 1950s. In a historical cohort study that represented an important advance in epidemiological methods, in addition to providing important new data about asbestos, Doll (1955) [2] reported a more than 10-fold excess of lung cancer among British asbestos textile workers. In the ensuing years, numerous epidemiologic studies of both workers exposed to asbestos in several industries and individuals with environmental exposure to asbestos have been conducted in countries around the world [3]. Collectively, the accumulated studies provide data about exposure to several types of asbestos fibers in diverse settings and report positive associations with cancer at several sites.

Asbestos was one of the first agents evaluated by the newly-formed International Agency for Research on Cancer (IARC) Monographs program in 1972 [4]. Formal evaluation statements were not yet used in the Monographs, but the Working Group concluded that all commercial forms of asbestos could induce mesotheliomas and lung tumors in experimental animals exposed by injection or inhalation. The epidemiological evidence available at the time largely concerned observations of increased risk of mesothelioma and lung cancer among workers in industries that manufactured or applied asbestos products. In summarizing that evidence, the Working Group noted that cancer risks appeared to vary depending on the type of fiber or by industry, with apparently lower risks for chrysotile.

The carcinogenicity of asbestos was evaluated by a second IARC Working Group in 1976 [5]. The conclusions from experimental studies with animals remained largely the same, and the summary of human evidence stated explicitly that exposure to chrysotile, amosite, crocidolite and mixed fibers increased the risk of lung cancer and mesothelioma. Increased risks of cancers of the larynx and gastrointestinal tract were also noted for the first time.

The U.S. National Toxicology Program also evaluated the carcinogenicity of asbestos around the same time. Drawing largely on the same studies evaluated by IARC, asbestos was listed in the First Annual Report on Carcinogens, issued in 1980, as “known to be a human carcinogen” [6].

An update of previous IARC evaluations of asbestos was reported in a condensed format in 1987 [7]. By this time, formal evaluation statements had been introduced in the Monographs and the Working Group concluded that there was sufficient evidence for the carcinogenicity of asbestos in humans and in experimental animals, classifying asbestos in IARC Group 1, carcinogenic to humans.

The most recent and comprehensive evaluation of the carcinogenicity of asbestos was conducted in 2009 for Volume 100 of the IARC Monographs [3]. The literature had grown significantly by that time, and the IARC Working Group now concluded that there was sufficient evidence that “all forms of asbestos”, specifically including chrysotile, crocidolite, amosite, tremolite, actinolite and anthophyllite, are carcinogenic. The evidence of a causal relationship was judged to be sufficient for mesothelioma and cancers of the lung, larynx and ovary and limited for cancers of the pharynx, stomach and colorectum. The evidence of carcinogenicity in experimental animals was also judged to be sufficient for the same six forms of asbestos. A striking feature of this last evaluation is the accrual of additional human cancer sites with sufficient or limited evidence of a causal association. Whether or not the risk of cancer varies among different types of asbestos was re-examined using the larger body of data now available. With respect to lung cancer, the Working Group concluded that it was “not possible to draw any firm conclusions concerning the relative potency of chrysotile and amphibole fibres”. In contrast, for mesothelioma, the Working Group judged that there was “considerable evidence” of differential potency among asbestos fiber types, with chrysotile having lower potency then amphibole asbestos. However, differences between studies with respect to exposure situations, industries and study methods, particularly methods of exposure assessment, were noted as other factors potentially contributing to heterogeneity in the risks observed in different studies [3].

A number of studies published since the last IARC evaluation lend further support to longstanding conclusions [8,9,10,11]. Important new data relevant to some outstanding questions have also been added. A study of Chinese asbestos miners and textile workers exposed to essentially pure chrysotile reported statistically-significant positive trends in lung cancer mortality with increasing cumulative exposure; notably, among miners, who appeared to have lower risks in some older studies, the smoking-adjusted relative risk per unit of exposure was somewhat higher than among the textile workers (1.20 compared to 1.12 per 100 fiber-years/mL) in the recent Chinese study [12]. Other pertinent Chinese studies on chrysotile are described below in Section 4.

Increased risk of cancers of the digestive system, including the esophagus, stomach and colorectum, were also reported in several studies. All three cancers were in excess in a general-population cohort in the Netherlands [13] and among Chinese chrysotile miners, with significant trends for esophageal and stomach cancer in the latter [14]. Elevated risk of colorectal cancer, but not esophageal cancer, was also reported in a study of workers at a factory in France that used both chrysotile and amphibole asbestos [8]. The evidence of these associations was evaluated as “limited” by IARC, meaning that the causal interpretation is credible, but the role of bias or confounding cannot be excluded.

The question of relative potency was examined in a meta-analysis of 19 studies that assessed the influence of study quality parameters on exposure-response estimates for asbestos and lung cancer [15]. After accounting for the quality of exposure assessment, there appeared to be little difference in the slopes for cumulative exposure to chrysotile compared to amphibole fibers. A recent British study quantified fiber lung burden in mesothelioma patients showing results for transmission electron microscopy (TEM) counts above 0.05 million fibers ≥5 µm in length per dry gram of lung tissue in 76% of the male cases. They confirmed that most mesotheliomas are caused by asbestos, even in the absence of an exposure history. The fitted model risk for mesothelioma was proportional to the amphibole lung burden. Chrysotile was virtually absent in the analyzed samples, and its proportional effect could not be estimated [16].

In conclusion, more than 40 years of evaluation have consistently confirmed the carcinogenicity of asbestos in all of its forms. New evaluations have served to expand the list of cancer sites associated with sufficient evidence to exposure to asbestos, beyond lung cancer and mesothelioma. Data from recent studies seem to support the possibility that colorectal cancer could be added, as well, if a new evaluation were conducted. On the other hand, early indications of an important differential in the risk of lung cancer according to the type of fiber have not been supported. Whether such a differential exists for mesothelioma is still an open question, which will be more difficult to answer because of the rarity of mesothelioma, the short half-life of chrysotile in the lung tissue and the absence of data reporting on fiber burden in the pleura.

## 3. Asbestos as a Global Threat to Health

According to recent figures, the annual world production of asbestos is approximately 2,000,000 tons, less than half the world production of 1977 (4,793,451 tons). This decrease is in large part explained by Canada’s reduction and finally stopping of asbestos extraction. Currently, about 90% of world asbestos comes from four countries: Russia, China, Brazil and Kazakhstan. Approximatively fifty percent of world asbestos is used by two countries, China and India, followed by Brazil, Indonesia and Russia. To date, total or partial asbestos ban legislation has been adopted by 54 countries. For more details, see Collegium Ramazzini 2015 [17], Marsili and Comba (2013) [18] and the site of International Ban Asbestos Secretariat (IBAS) (www.ibasecretariat.org).

Occupational exposure to asbestos causes an estimated 107,000 deaths each year worldwide [19]. The aforementioned Collegium Ramazzini statement summarizes estimates of the global burden of disease caused by asbestos. When the global burden of each type of asbestos-related disease (ARD) was considered separately, the estimated number of deaths per year was 41,000 for asbestos-related lung cancer (ARLC), 43,000–59,000 for mesothelioma and 7000–24,000 for asbestosis. In terms of prevention and public health policy, the Collegium Ramazzini has stated that in industrialized countries, large quantities of asbestos remain as a legacy from past construction practices in many thousands of schools, homes and commercial buildings. Significant quantities of asbestos also remain in various industrial applications. As the materials weather, erode, break or are cut by power tools, asbestos fibers are released into the air, soil and water, where they become a source of community-wide exposure. Policies, regulations and practices should safeguard workers engaged in the removal of asbestos-containing structures and the handling of the resulting waste material, via schemes for specialized training and licensing. Many industrializing countries have been slow to reduce, let alone ban, the use of asbestos. The multiple factors at play include the low price and easy accessibility of asbestos, demand from the construction sector in emerging economies, the scarcity of medico-social resources and sustained efforts by the asbestos industry to minimize the health risks related to asbestos and other parties who may be in a situation of conflict of interest.

Finally, the Collegium emphasizes that the accumulated wealth of experience and technologies in industrialized countries should be shared internationally through global campaigns to eliminate ARDs. The knowledge and technological developments that have emerged from prevention-oriented initiatives in industrialized countries could be of great benefit to countries in which asbestos continues to be used. Scientific expertise is an important resource to be shared, including capacity building and surveillance of ARDs. Given the wide range of problems encountered at the global level, the development of regional initiatives should be particularly valuable.

In light of the abovementioned issues, two specific points deserve particular attention.

Firstly, one is the integrity of the scientific literature with reference to attempts of improper penetration by scientists who have different levels of associations with the asbestos industry. Ilgren *et al*. (2015) [20], for example, recently published a “critical reappraisal” of the Italian study on mesothelioma among workers and residents in the Balangero site where chrysotile was extracted for many decades. The Ilgren paper contained many errors and was the object of a rebuttal by Magnani *et al*. (2015) [21] followed by the publication of an erratum by the journal [22]. The same authors [23,24] also tried to demonstrate the lack of carcinogenicity of Bolivian crocidolite, again with clearly inappropriate procedures and flawed arguments. Even if easily confuted in the scientific setting [25], these papers may, either internationally by lawyers or a result of scientific illiteracy on the part of lawyers and/or judges, attribute unwarranted consideration and weight.

Secondly, scientists have a role in serving the court as expert witnesses. The role of an expert witness is to serve the court, not serve the interests of plaintiffs or the defense, a rule that is, admittedly, frequently breached. It does also happen that the courts prescribe *ad hoc* epidemiological studies. An example of such a court-ordered study is the case of the Eternit plant in Bagnoli (Naples) where a cohort mortality study (1965–2005) was conducted in order to ascertain if there was a significant excess of asbestos-related disease in a given factory or urban district. As a result of the study, significant excess risks were found in asbestosis, malignant pleural neoplasm, lung cancer and peritoneal cancer [26]. The judicial context also provides the opportunity for both plaintiffs and defense to refute “bad” science presented to the court by the other side. Tomatis *et al*. (2007) [27] discuss cases where asbestos industry defense lawyers and consultants have used questionable evidence (e.g., absence of dose-response relationships; lack of risk of inhalation of fibers other than that associated with ultra-thin and ultra-short fibers; risk confined to very early exposures; risk confined to amphiboles; and the notion that there is a “safe” use of asbestos). Conversely, it is in the experience of the authors that in given circumstances, plaintiffs and their lawyers have erroneously attributed to asbestos exposure cases of diseases for which the evidence of a causal role of asbestos was inadequate or absent. To be clear on this last aspect is a deontological requirement in order to pursue a really equitable trial for authentic asbestos victims.

In fact, there is an overwhelming amount of scientific evidence to support a ban on the use of asbestos. Even in countries banning asbestos, asbestos-related disease will continue to occur as a result of asbestos that remains in pre-existing buildings and products and due to the fact that the latency period for asbestos-related disease to be diagnostic is typically 10–40 years or more following exposure.

Global drivers of economy still operate in the direction of encouraging production and use of asbestos, especially in Russia, Asia, Africa and Latin America. Their strategy includes pressures on governments, workers and labor unions, attempts to penetrate the scientific community, cover up conflicts of interests in research and manipulation of media information [17,18].

International scientific cooperation towards the fight against the extraction and use of asbestos can contribute to the prevention of asbestos-related disease as a fundamental strategy. In this framework, the accumulated wealth of experience and technologies in industrialized countries should be shared internationally and inspire the developments of approaches well tailored to other unique contexts.

## 4. Arguments to Justify the Ongoing Use of Asbestos: Are They Correct?

Currently, approximately 80% of the global population lives in countries were asbestos has not been banned.

The Chrysotile Institute [28] is one of the most important industry-supported international institutes promoting the use of chrysotile asbestos. The Chrysotile Institute claims to have organized more than 100 missions in 60 countries to “take part in international conferences by offering relevant documentation, advice, or technical, medical and scientific training to chrysotile producers and users of chrysotile in other countries as well as to industrial health specialists” [28]. The principal arguments put forth by industry to promote the use of asbestos are that: there is a potency difference between chrysotile and other more potent forms of asbestos; non-friable products prevent exposure to asbestos; substitutes for asbestos can also cause disease; and that it is possible to control and use asbestos safely.

- Chrysotile asbestos is less potent than other asbestos forms: The Chrysotile Institute states that “there is an overwhelming body of evidence, based on epidemiological studies on clinical findings, and on lung tissue mineral analysis in humans showing a definite difference in potency between chrysotile and the amphiboles” [29]. It is important to highlight several issues in this regard:
  (a) The International Agency for Research for Cancer (IARC) has repeatedly classified all forms of asbestos, including chrysotile, as carcinogenic to humans (Group 1, known human carcinogens). Asbestos exposure causes asbestosis, mesothelioma and cancer of the lungs, larynx and ovaries [3].
  (b) The Chrysotile Institute selectively cites four studies to support its arguments for a potency difference [30,31,32,33], while ignoring the growing body of recent evidence of chrysotile-related disease in studies conducted in China involving textile and mining workers [12,14,34,35,36,37,38]. These recent studies show evidence of excess risk for mesothelioma, lung cancer and digestive cancers in workers exposed to chrysotile. On a quantitative basis, these new findings have not yet been fully analyzed in order to improve the assessment of the potency difference between chrysotile and other forms of asbestos. Furthermore, for the specific case of asbestos and applying a preventive approach [39], controlling the use of this carcinogen should not be based on the comparison of the carcinogenicity of chrysotile asbestos against amphibole asbestos.
  (c) The Chrysotile Institute states that “Only high-density chrysotile products are manufactured and sold today. The unique feature of these products is that the chrysotile fibre is encapsulated in a matrix of cement or resin, preventing the release of fibres. Over 90% of chrysotile used worldwide today is in the manufacture of fibre-cement building and construction materials” [40]. This notion that asbestos-containing products that are non-friable prevent asbestos exposure is a misleading statement because disease-causing asbestos fibers can be realized even from high density chrysotile products when these products are manipulated. Most of the manipulation of the so-called high density chrysotile products comes from construction, where sawing, drilling and adjusting asbestos-cement products are daily practice. Breathable asbestos fibers are generated in these operations. Construction workers in industrializing countries are completely unaware of asbestos exposure. Recent evidence collected in Iran and Colombia clearly documents high chrysotile asbestos exposures among auto mechanics that manipulate non-friable asbestos-containing products [41,42,43,44]. Furthermore, in the case of auto mechanics, it has been shown that the manipulation occurs because of the physical form in which both brake and transmission asbestos-containing products are commercialized by the asbestos industry, since they are sold detached from the supports needed for the installation in the vehicles. Thus, mechanics are forced to manipulate brake and transmission asbestos-containing products in the shops, which expose them to the fibers [42,43,44]. The risk of exposure in other occupations has also been recently investigated. A study in Iran [45] showed that during demolition of old houses in Tehran, workers were exposed to high chrysotile asbestos concentrations. Since asbestos containing construction products are currently used in many countries in the world, a future risk of asbestos exposure during eventual demolitions of these buildings has been and is currently being created. Furthermore, it should be considered that the encapsulating material of asbestos-containing products will eventually deteriorate, and asbestos fibers will be released from these asbestos-containing products that have been and are currently distributed worldwide.
- Questions raised regarding the toxicity and safety of asbestos substitutes: The Chrysotile Institute states that “Replacing chrysotile is a very complex operation. Evaluations of the risks and hazards of a good many other fibres are now clear enough that legislators are beginning to impose regulations to control these substitutes. In 1993, a group of experts brought together by the World Health Organization (WHO) issued Environmental Health Criteria 151, stating that all respirable and bio-persistent fibres must be tested to check their toxicity and carcinogenicity. In fact, recent studies have shown that many fibres used to replace asbestos in numerous products may be as hazardous or even more hazardous than chrysotile asbestos: this is notably the case for fibreglass, rock wools, refractory ceramic fibres and aramid fibres. In 1993, the International Program on Chemical Safety (IPCS) explicitly recommended that exposure to any respirable and durable fibre be controlled to the same extent as that required for asbestos until the data prove that lesser controls would be sufficient” [46]. It is true that before proposing a substitute for asbestos, the hazard of the substitute should be assessed. However, the WHO [47] organized a workshop with experts in 2005 (*i.e.*, 12 years later than Environmental Health Criteria 151 cited by the Chrysotile Institute), and this group of experts identified several substitutes for asbestos that could be classified as being a low hazard for humans. These low hazard substitutes include short fibers of attapulgite, carbon fibers, non-respirable cellulose fibers, non-bio-persistent wool-like synthetic vitreous fibers, natural wollastonite and xenolite [47]. Thus, safer substitutes for asbestos have been identified.
- The argument that a controlled or safe use of asbestos can be achieved: The safe or controlled use argument is usually provided in a hypothetical scenario, without hard evidence to support it. For example, the Chrysotile Institute states that “The chrysotile industry created and is now implementing a responsible-use programme that is based on the controlled-use approach to regulating chrysotile” [48]. No evidence is provided to support that safe or controlled use is being achieved or is achievable at the global level in all of the countries that still use asbestos-containing products. In fact, high exposures to asbestos, similar to those found in the past, can still be observed today in auto-mechanics that manipulate asbestos-containing products [42,43,44], providing evidence in a specific occupation that safe or controlled use is not being achieved. Thus, a valid question would be if the lack of safe or controlled use observed in different sectors, such as in auto mechanics shops, construction, demolitions and dismantling of ships, could also be found in other occupations that have to work with asbestos-containing products. Furthermore, the WHO has emphasized the difficulties to achieve controlled use in other sectors, such as construction [49]. Scientists, governmental officials and the public in general should exert caution in the face of arguments put forth by the asbestos industry to justify the use of the material. International agencies, such as the WHO and the International Labour Organization (ILO) (among others), state that the most effective way to prevent asbestos exposure and prevent asbestos-related diseases is to end the use of the material [49,50,51].

## 5. Epidemiological Studies on Asbestos-Related Diseases

In many countries that still use asbestos, working conditions do not even meet the standards for limited protection of workers, which were adopted in traditionally industrialized countries before the asbestos ban. Preventive measures are urgently needed even in the absence of ”local” estimates of the health loss caused by asbestos: knowledge on the hazardous effects of asbestos is detailed enough as to allow an extrapolation of estimates obtained elsewhere. However, new, “local” studies are desirable for several reasons. In the first place, they can stimulate awareness of the size of the problem by public opinion and other stakeholders. Further, they can provide important information on the circumstances of exposure, as well as local asbestos-related health impacts. In these contexts, studies to be carried out should be identified considering at least three key aspects: (1) the level of interest of results, distinguishing between the national and the local level (*i.e.*, evaluating where and how results from the studies can be useful for preventive strategies); (2) the strength of results in terms of usefulness in developing preventive interventions; (3) the economical and technical feasibility of the studies. In most countries that still use asbestos, local estimates of the impact on health of asbestos are scarce. This is, for example, the case of the four Latin American countries that are or have been the highest asbestos consumers in the region: Argentina, Brazil, Colombia and Mexico [52]. No more than a handful of descriptive and analytical epidemiological (case-control or cohort) studies on asbestos-related cancer have been reported from these countries in the open literature: most likely what they describe represents the tip of an iceberg.

The relevance of results from local epidemiological studies can be inferred considering the “Outline for the development of national programmes for elimination of asbestos-related diseases” (ARD) proposed by the WHO together with the ILO [50]. According to this document, the main domains for collecting information are: local asbestos use, its presence in the general environment, health-related impacts (*i.e.*, the magnitude of the problem) and evaluation of the asbestos-related economic and social aspects. Defining the magnitude of the problem requires: (1) collection of information on the production, import and export of asbestos; (2) identification of exposure sources, starting from the industrial activities entailing the use of asbestos and asbestos-containing materials; (3) estimate of the probability of the occurrence (and levels) of potential exposures; (4) qualitative and quantitative assessment of health risk in different contexts and the asbestos-related burden of disease.

Worldwide, a number of countries face the need to quantify the health loss brought about by asbestos, but lack adequate information systems. For a complete assessment of the asbestos health impact, all diseases with a known causal association with asbestos exposure (*i.e.*, asbestosis, pleural plaques, mesothelioma and cancers of lung, larynx and ovary) should be considered. Nevertheless, the study of mesothelioma offers several advantages: the association with asbestos is very specific (*i.e.*, in practical terms, there are no other risk factors); there is no safe threshold of exposure; and the risk is proportional to the cumulative dose of exposure [53].

The first approach to be suggested is the simple collection of mesothelioma cases with the description of their age, sex and place distribution and the reliability of diagnosis. Whenever possible, data on each case should be supplied with anamnestic information regarding asbestos exposure. The latter point requires caution since poor anamneses mean loss of sensitivity in the identification of asbestos exposures. This kind of data collection does not allow a quantitative risk estimate, but it can be useful when the asbestos risk (and the need for prevention) is denied with the excuse that data “are uncertain”. At a national level, the asbestos health risk can be estimated using a well-established function between mesothelioma mortality and previous asbestos consumption [54]. This approach uses a predictive model of risk based on real data on asbestos consumption. It provides an estimate of the risk of mesothelioma mortality also in the absence of any data about the outcomes. Another approach is based on routinely-collected data on vital statistics. Since mesothelioma is a lethal disease with a median survival time close to one year, indicators of asbestos risk can be obtained using mesothelioma mortality data. Such an approach was used to estimate the incidence of mesothelioma and to evaluate temporal trends and spatial patterns of asbestos risk at national levels [55,56]. It requires a preliminary validity assessment of mesothelioma mortality data for population-based studies [57].

For epidemiological surveillance, population-based cancer registries have, at least in principle, the advantage of a better validity of data on diagnosis, even if the validity should be verified specifically for mesothelioma. Nevertheless, population-based cancer registries are available only in some countries, and their coverage is often local and not national [58].

The indicator “population attributable fraction (PAF)” can be used to estimate the proportion of asbestos-related diseases that would be preventable through the elimination of exposures. PAF can be calculated using relative risks obtained from population-based analytical studies (case control or cohort) and estimates of the prevalence of persons exposed to asbestos in the population. In the absence of analytical studies, in a given population, a proxy to PAF can be estimated on the basis of estimates of asbestos use in the target population and some assumptions on relative risks. This approach was used to estimate, at the national level in some Latin American countries, the burden of cancer associated with asbestos exposure in the occupational context [59]. Mesothelioma population-based registries were created in some countries [55]. They collect information at the individual level, both on diagnostic reliability and on asbestos exposure. This kind of information can be used to identify strategies for improving diagnostic capabilities and to define occupational and environmental contexts at major risk. Data from these registries can be used to identify priorities for remediation interventions, preventive activities and health surveillance programs. Nevertheless, these registries require considerable economic resources and the availability of skills in occupational and environmental hygiene.

Finally, epidemiological analytical studies, in particular those with a cohort and case-control design, can be carried out to better estimate asbestos risk in specific populations, both in occupational settings and in populations environmentally exposed to asbestos [60]. Their results can also be used for risk analysis at the national level [61]. The inferences that can be made from analytical studies depend on the validity of the assessments at the individual level, both on asbestos exposure and on health outcomes.

## 6. Epidemiology and Prevention of Asbestos-Related Diseases in Brazil

Brazil is one of the five largest worldwide producers, consumers and exporters of asbestos [62]. This economic activity that started in the 1940s grew more intensively during the 1970s, reaching the peak between 1985 and 1991. While consumption of asbestos-containing materials is widely distributed across the country, the asbestos extraction is currently concentrated in the State of Goiás in the Middle-West Region. Over 98% of the asbestos consumption is for asbestos-cement manufacturing. Until 2007, the State of São Paulo harbored approximately half of the asbestos industries, with the remaining distributed in the eastern regions of the country [63]. From the 1960s to now, over 6,000,000 tons of asbestos have been used throughout its production chain [64,65]. Asbestos-cement products, mainly roofing and other construction materials, are estimated to be used in 40%–50% of Brazilian buildings [66]. Costa *et al*. [67] estimated that in 1980, there were 20,000 workers from 120 asbestos firms in the country, 12,000 workers in the asbestos-cement industry, 3000 in the friction products manufacturing, 3000 in asbestos textile production and 2000 in mining and processing plants.

Studies on the health effects of asbestos in Brazil started early in the 1950s. The first report, released by the late Ministry of Labor, Industry and Commerce, described 16 chrysotile miners out of a group of 65 from the state of Minas Gerais [68] whose X-ray exams were compatible with asbestosis. Other case studies depicting asbestosis, pleural plaques, asbestos-related lung cancer and mesothelioma were also published [69,70,71]. Although simple in design and analyses, they played a relevant role in demonstrating that in Brazil, asbestos also caused health problems. Other case studies were based on compensation benefits granted by the Social Insurance Institute [67,72].

Morbidity and mortality estimates appeared later. A study of workers from three asbestos-cement industries in the state of São Paulo estimated a 10.1% prevalence of X-rays compatible with asbestosis when the analysis was limited to those with 10 or more years of tenure at their jobs [73]. A larger observational study of 828 former asbestos-cement workers estimated 29.7% and 8.9% cases of pleural thickening and asbestosis, respectively, and demonstrated lower values of lung function associated with increased quartiles of semi-quantitative exposure [74]. Part of this group was followed in a cohort study that found occupational asbestos exposure as a risk factor for lung function decline [75]. Another investigation in Brazilian chrysotile mining and milling showed that the risk of non-malignant lung abnormalities and lung function impairment declined over time as asbestos exposure was progressively reduced [76]. This conclusion has limitations because of the short time of observation of workers recently hired by the mine.

In a hospital-based case-referent study on occupational risk factors of lung cancer in the city of São Paulo, no significant risk was detected for asbestos exposure, assessed through lifetime job history [77]. The absence of risk was possibly due to cases and controls being interviewed too early following the expansion of asbestos consumption. Other studies provided mesothelioma mortality estimates in Brazil based on the Mortality Information System (SIM). From 1980–2003, the overall mesothelioma mortality was estimated at 0.56/1,000,000 in 1980, increasing to 1.01/1,000,000 in the last study year, for all ages, corresponding to an 80.4% growth [78]. Algranti *et al*. [79] analyzed mesothelioma deaths from 2000–2012, for adults 30 years of age or older, and reported that in spite of an increasing time trend in age-adjusted standardized mortality found in the state of São Paulo, there was no statistically-significant changes for the entire country. Clusters of mesothelioma cases were found in municipalities with asbestos-cement industries. Forecasting estimates suggested that the mesothelioma mortality will grow until the next decade, with a mortality peak around 2021–2026 [79]. A study conducted with data from Rio de Janeiro found 224 death certificates with International Classifications of Diseases ICD-9163 (cancer of the pleura) as the underlying cause of death. The authors matched the cases with histopathology data and medical records, disclosing confirmation rates of only 35.5% and 59.3%, respectively [77,80].

Estimates of asbestos consumption in Brazil were close to 1.6 kg/per capita in 1991 when they started to steadily drop, reaching a bottom figure of 0.6 in 2003; at this year, it started to raise again, until it reached 1.0 kg/per capita in 2012. Mining, milling and uses of amphiboles have been forbidden since 1991. Currently, a federal law permits mining and milling, processing and uses of chrysotile asbestos in Brazil, and six Brazilian states legally prohibited the manufacturing and use of asbestos products. These laws enforcements, together with the increasing awareness about the asbestos health effects, resulted in a reduction of internal consumption, whereas exports to industrializing countries augmented [62] (in Brazil, to date, the States of São Paulo, Rio de Janeiro, Amazonas, Pernambuco, Rio Grande do Sul, Minas Gerais and Mato Grosso prohibited asbestos). The asbestos industry stakeholders allege that the state laws contradicted the Brazilian federal law, opening space for controversies and unfavorable legal decisions regarding workers’ health protection. In 2009, the Supreme Court made a provisional decision favoring the ban of asbestos in the state of São Paulo, based on the evidence that it is an occupational, public health and environmental risk that threatens a citizen’s constitutional right to a healthy and safe work environment. Later in 2012, asbestos hearings were held at the Brazilian Supreme Court to inform the Court on the issues involving work and health, environment, asbestos substitutes and economic issues, but no final decision was released until now [81].

There is a written agreement between two workers’ confederations, the workers’ commission from the single chrysotile mining plant, both partially funded by the asbestos industry, the national confederation of asbestos-cement industries and the national confederation of industries to adopt a fiber exposure limit of 0.1 fiber/mL among other preventive measures [82]. However, public statements from the Ministry of Labor and Employment and the Health Ministry at the Brazilian Supreme Court were favorable to the asbestos ban, also supported by the IV National Conference of Workers’ Health held in December 2014, an assembly that congregates delegates from all labor unions, associations and movements.

In summary, there is evidence on the occurrence of non-malignant asbestos-related diseases, similar to what has been described in the 1970s and 1980s in industrialized nations. There are clear signs of an increase in mesothelioma mortality in the state of São Paulo, where almost half of the asbestos industries were settled and specialized healthcare services, able to provide better diagnosis resources, are available. At the present time, the fate of asbestos in Brazil is unclear. If the Supreme Court rules against the ban already addressed in state laws, it is probable that both internal consumption and asbestos exports will increase, expanding the local and world health burden of asbestos-related diseases.

## 7. Disease Experiences in Asbestos-Contaminated Communities

There is growing concern, both in the scientific community and in the sphere of public opinion, for the social and psychological impact of asbestos in communities that experienced elevated occupational and environmental levels of exposure. This issue can be addressed by investigating to what extent the strategies elaborated by sufferers in order to face the effects of asbestos contamination on their own bodies, as well as on their affective spheres can promote relevant changes in the surrounding socio-political context, inexorably linked to the global economic and political dynamics of asbestos.

Such a framework has been adopted by anthropologists who did their research on disasters [83] and in areas affected by catastrophic events [84,85,86]. The consequences of asbestos contamination on an exposed population can be considered as disasters, even in the absence of a judicial acknowledgment or epidemiological evidence. In fact, by considering any disaster as a process [86], in some contexts, the destructive impact on a population can be slow, unperceived and invisible. Slowness and invisibility in fact are specific characteristics of disasters caused by asbestos pollution [87,88,89,90,91,92]. In this regard, the invisibility occurs in relation to the political-economic, cultural, and social dynamics at stake in asbestos related illness experiences, for instance in the difficulties to achieve a correct diagnosis for ARDs and the consequent inadequate registration of **asbestos related** deaths and diseases [59,91,93]. The invisibility characterizes also the practices and management of risk perception concerning asbestos carcinogenicity [87,94]. However, in some contexts, AR disasters can turn visible and can be recognized, for instance, by established biomedical and judicial institutions, despite the contrasting pressures of asbestos lobbies, particularly aggressive in countries where asbestos is still legal [92].

Studies based on this approach have been performed in the Italian urban settings of Bari and Casale Monferrato (Italy) [89,90] and are currently ongoing in Osasco, São Paulo (Brazil).

Osasco is a city with 700,000 inhabitants with a history of massive industrialization, as well as of relevant socio-political and cultural struggles, often related to the campaigns organized by the strong trade unions that were, and still are, based there [95,96]. In Osasco, a group of workers who, during their professional life, had been exposed to asbestos, began mobilizing in the beginning of the 1990s, on the basis of an increasing number of them getting sick from respiratory diseases. Starting from noticing the disaster’s effects on their own bodies, they began mobilizing and building a tight dialogue with local biomedical doctors, public officers, political groups, trade unions and lawyers engaged in the anti-asbestos movement. This last aspect situates the practices the sufferers undertook, in activism addressed to achieve the biomedical, epidemiological, social and judicial recognition of the suffered violence [97,98,99,100,101]. To this concern, points of contact between the strategies undertaken by the activists from Casale Monferrato and Osasco can be found. The practices applied in Osasco appear to be emblematic examples of the strategies of the struggle undertaken by the activists in order to make visible the disaster from which they are suffering. These practices were undertaken by the activists of the ABREA (Associação Brasileira dos Expostos ao Amianto (Brazilian Association of Exposed to Asbestos), and research collaborators were former workers with asbestosis and pleural plaques, families of women and men who had died because of malignant mesothelioma and by subjects aware of their own risk of contracting a fatal cancer because of previous, occupational and/or environmental exposure to asbestos. By referring to anthropological and psychological studies on communities affected by a disaster [85,102,103], all of them can be defined as sufferers of the consequences of asbestos manufacturing.

In this socio-political context, the ABREA, founded in 1995, became one of the principal actors in Brazilian anti-asbestos mobilization and a primary reference for the Virtual Citizens’ Network for the Banning of Asbestos in Latin America [92,104,105]. Moreover, the ABREA is actively situated in the global scenario of anti-asbestos activism, with particularly strong social, historical and affective connections to the anti-asbestos movement organized by the victims from Casale Monferrato in Italy. During twenty years of civil struggles, the ABREA played a fundamental role in raising awareness about the dangers of asbestos and promoting asbestos bans by constant participation in the socio-political, biomedical, juridical and legislative discussion on asbestos carcinogenicity.

In this context, a final point deserves specific attention. By referring to anthropological, philosophical and sociological studies on health-based social movements [100,101] and on the relations between power and body [106,107], attention can be drawn to the bodily dimension of the investigated socio-political and cultural phenomenon [108,109]. From this perspective, the socio-political engagement is triggered by the crisis provoked by the sufferer’s experiences (e.g., AR illness, risk and grieving experiences) that can disrupt the world one is used to [110]. Such a crisis, representing a condition from which new practices and meanings can be promoted, can flow beyond the individual body and one’s closest affective world and invest the surrounding context. In this way, a potential of critique and reflection comes to interest the processes through which new knowledge is elaborated and decisions are made, at both local and global levels.

## 8. The Role of International Cooperation in Fostering Public Health Response to Asbestos: The Experience of the Italy-Latin America Network

As demonstrated in previous sections, asbestos still represents a global threat. For this reason, international scientific cooperation can play a relevant role in fostering public health response to asbestos by strengthening the prevention of asbestos-related diseases in those countries where asbestos is still permitted and widely used. Two major lines of evidence support such an international effort. Firstly, asbestos represents a public health issue both in countries that banned asbestos after producing and/or using asbestos for many decades and in those countries where asbestos is still widely used today. This is due to the great amount of asbestos produced and used, as well as the long latency of the asbestos-related disease. Secondly, scientific cooperation is a suitable framework to facilitate the transfer of knowledge, as well as exchanges of experiences matured in different countries on various issues concerning asbestos and asbestos-related disease within and outside the health domain.

A critical requirement for establishing an effective international cooperation framework is the adoption of a bidirectional approach to exchange expertise and experiences among collaborating scientists and institutions of different countries. This is mandatory in order to meet local needs and priorities related to asbestos issues. Multidisciplinary skills have to be included in the cooperation framework through a common vision dedicated to promote:

- new epidemiological studies for assessing the health impact of asbestos in specific contexts;
- socio-cultural and economic analyses for contributing to identifying stakeholders and to address both local and global implications of asbestos diffusion;
- public awareness on the health and socio-economic impact of asbestos use and banning.

The Italy-Latin America cooperation network presented in the present article has adopted this vision and approach. Scientists from academic and research institutions of Italy and Latin American countries are collaborating to foster epidemiological research and literacy on the prevention of asbestos exposures in working and living environments and asbestos-related disease. The cooperation initiatives include the mutual exchange of skills and experiences, collaborative studies, training and dissemination activities in Latin America, where asbestos is still widely used. In these countries, a few epidemiological studies have been performed so far, and more efforts are needed to increase awareness on the asbestos health impact [52,59].

Training and dissemination activities represent an essential contribution to this international cooperation framework. Dissemination relying on validated epidemiological and social research on asbestos contributes to identifying and providing appropriate tools to local and national stakeholders for strengthening awareness on asbestos issues. This can foster an informed decision-making process for adopting health prevention actions and inter-sectorial interventions. The diversity of the stakeholders involved in the initiatives performed in this cooperation framework—scientists, health professionals, health and environmental authorities, associations of exposed workers and non-governmental organizations is an essential element to promote a prevention culture.

The adoption of a public health approach for the prevention of asbestos-related disease by this cooperation network can contribute to tackling environmental health inequalities associated with an unequal distribution of hazardous exposures within and among countries [111,112]. Health inequities concern the preventable burden of asbestos-related diseases in those countries where asbestos is still used with respect to those that adopted a prevention policy and banned asbestos in the past decades. It is important to point out that health inequities affecting population subgroups or countries are related to the social-economic determinants of health [113,114,115]. For this reason, health inequities related to asbestos require a broad socio-economic analysis, including issues outside the health domain at the international and local scale (e.g., international trade regulation, economic relationships between asbestos exporting/importing countries, industrial development and labor policies, education and literacy).

The Italy-Latin America cooperation network, involving epidemiologists and social and environmental health scientists, is open to develop collaborations and initiatives in countries where asbestos is still used to promote capacity building and epidemiological studies. Initiatives aimed to increase preparedness and awareness of affected communities have to engage representatives from society, such as local associations, which can play a primary role for the elimination of involuntary/unaware hazardous exposures in working and living environments.

The cooperation framework relies on sharing knowledge concerning occupational and environmental epidemiological studies on asbestos in Latin America and in Italy, as well as on performing joint health promotion initiatives targeted to different stakeholders in Latin American countries. These initiatives also focus on enabling the aware use of validated documentation and sources on asbestos issues, such as statements edited by international organizations (WHO, IARC, UN Rotterdam Convention, ILO, International Commission on Occupational Health, Collegium Ramazzini) on the prevention of asbestos-related disease and asbestos banning. The performed training and dissemination activities in Latin American countries have involved academic institutions, local and national health authorities and NGOs from Colombia, Ecuador, Bolivia and Brazil [116,117].

In this cooperation framework, the participation in the 27th Conference of the International Society for Environmental Epidemiology (ISEE) held in Brazil (São Paulo) on 30 August–4 September 2015 “Addressing Environmental Health Inequalities” represented an important occasion to consolidate the cooperation network. In particular, the symposium “Prevention of Asbestos-Related Disease in Latin America: Reducing Inequalities by International Cooperation” organized by the Italian, Brazilian and Colombian cooperating partners was an opportunity to present experiences, share the outcomes of recent studies and plan future collaborations.

## 9. Conclusions

In light of the overwhelming scientific evidence on the health impact of asbestos that has been reviewed and discussed in the present paper and taking into account the societal values connected with environmental quality, health protection, equity and accountability, the following conclusive remarks appear warranted.

(1) The continuing use of asbestos is not compatible with the notion of sustainable development as defined by the UN Rio Declaration on Environment and Development (1992) and more precisely by the goal (No. 12) “Responsible Consumption and Production” within the 2030 Sustainable Development Agenda [118]. Industrial development based on asbestos use is economically viable only inasmuch as environmental and health costs are externalized.
(2) Even if a generalized ban of asbestos use were reached at a global level, many problems would still remain unsolved, both at the global and local levels, because of the widespread occurrence of asbestos in all of its forms in the industrial and urban environment and in the waste cycle.
(3) Some advocates in favor of asbestos use have disseminated inaccurate or misleading information in scientific journals and public fora. A major effort of public health is thus required in order to contrast the continuing use of asbestos at a planetary level, including refutation of false or misleading scientific claims.
(4) International cooperation between scientific institutions of industrialized and low- and middle-income countries, including low-intensity research countries, is required in order to effectively pursue a global prevention of asbestos-related disease, including highlighting environmental heath inequalities among countries.
